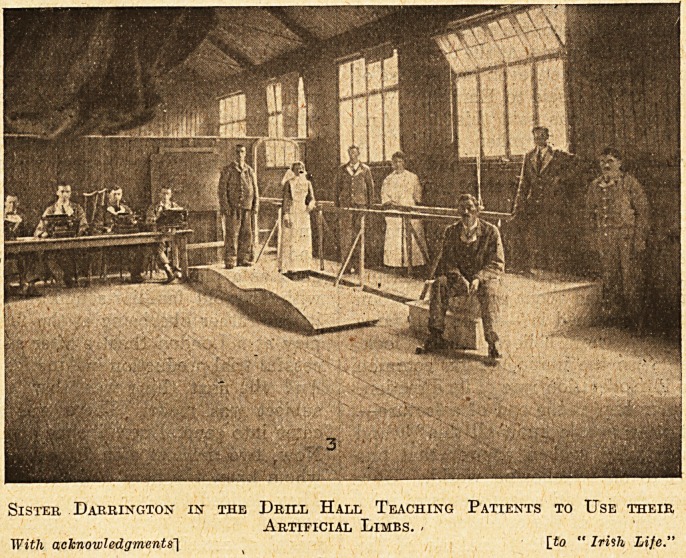# For Irish Sailors and Soldiers Who Have Lost a Limb

**Published:** 1917-10-06

**Authors:** 


### DUKE OF CONNAUGHT'S AUXILIARY HOSPITAL, BRAY.

### For Irish Sailors and Soldiers who have Lost a Limb.

Situated in the outskirts of Bray, Co. Wicklow, is the Duke of Connaught's Auxiliary Hospital for Irish sailors and soldiers who have lost one or more limbs. Formerly used as an industrial school, the building suits its present purpose admirably. It stands in extensive grounds and commands delightful views of sea and mountain. The hospital is, in its miniature way, fashioned after the model of Roehampton, but Captain de Burgh Daly, R.A.M.C., who is the C.O., claims that the artificial legs that are being used here are an improvement on those in vogue at Roehampton. In the first place they are only about half the weight; and, secondly, the "bucket," or thigh portion of the leg, is quite different in its make. The Roehampton model is shaped like a thimble, and into this the stump fits. The usual effect of inserting the stump into the artificial socket is to press the flesh upwards, with a resulting soreness. The "bucket" in use at the Duke of Connaught's Hospital is not a solid ring, but opens like a boot, and is closed by the process of lacing. The stump is inserted, the flesh pressed downwards, and the "bucket" is then laced from the top to the bottom. The Roehampton leg is "worked" from the man's shoulder—the Duke of Connaught's from the hip.

Both Miss Dowley, the matron, and Sister Darrington, who acted in her stead when the former was on leave, were previously employed at the 4th London Territorial Hospital. The staff consists of an assistant nurse and a number of V.A.D.s, some of whom are resident. There are sixty beds, and most of these are at present occupied, the patients appearing thoroughly to enjoy their stay at the hospital.

Sister Darrington in the Drill Hall Teaching Patients to Use their Artificial Limbs.

*With acknowledgments*] [*to "Irish Life."*

The object of the hospital, as Captain Daly explains, is to provide the best artificial limbs that the nation can produce, to train wounded men to use them, and, in addition, to teach them handicrafts so as to enable them to earn a livelihood on their discharge from the Army, which invariably follows their residence here. An important regulation is that a wounded soldier may at any time after his discharge be readmitted for the adjustment, repair, or renewal of his artificial limb. The men learn, amongst other things, typewriting, basket-making, and boot-making, whilst many of them attend the Bray Technical Schools, which are excellently equipped and conveniently situated. The hospital is under the control of a very influential committee, a section of which devotes its attention to providing suitable employment for the patients as they leave, a matter of the greatest importance for the men. H.R.H. the Duke of Connanught is patron.

## Figures and Tables

**Figure f1:**